# Numerical and experimental study of aerosol dispersion in the Do728 aircraft cabin

**DOI:** 10.1007/s13272-023-00644-3

**Published:** 2023-02-15

**Authors:** D. Schmeling, A. Shishkin, D. Schiepel, C. Wagner

**Affiliations:** 1 German Aerospace Center (DLR), Institute of Aerodynamics and Flow Technology, Bunsenstr. 10, 37073 Göttingen, Germany; 2 Institute of Thermodynamics and Fluid Mechanics, Technische Universität Ilmenau, Am Helmholtzring 1, 98683 Ilmenau, Germany

**Keywords:** Aircraft cabin, Aerosol dispersion, COVID-19, Mixing ventilation, Displacement ventilation

## Abstract

The dispersion of aerosols originating from one source, the ‘index’ passenger, within the cabin of the aircraft Do728 is studied experimentally using an aerosol-exhaling thermal manikin and in Reynolds-averaged Navier–Stokes simulations (RANS). The overall aim of the present study is the experimental determination of the aerosol spreading for the state-of-the-art mixing ventilation (MV) and to evaluate the potential of alternative ventilation concepts for controlling the aerosol spreading in RANS. For MV, the experiments showed that the ratio of inhaled to exhaled aerosol particles drops below 0.06% (volume ratio) for distances larger than two seat rows from the source. However, within a single row, the observed ratio is higher. Further, the dispersion is much weaker for a standing than for a seated index passenger. High air exchange rates and a well-guided flow prevent a dispersion of the aerosols in high concentrations over larger distances. Additionally, the positive effect of a mask and an increased air flow rate, and especially their combination are shown. In the complementary conducted RANS, the advantages of floor-based cabin displacement ventilation (CDV) which is alternative ventilation concept to MV, regarding spreading lengths and the dwell time of the aerosols in the cabin were determined. The obtained results also underline the importance of the flow field for the aerosol dispersion. Further, additional unsteady RANS (URANS) simulations of the short-term process of the initial aerosol cloud formation highlighted that the momentum decay of the breathing and the evaporation processes take place within a few seconds only.

**Supplementary Information:**

The online version contains supplementary material available at 10.1007/s13272-023-00644-3.

## Introduction

With regard to the COVID-19 pandemic, which strongly affected the public transport sector, the spreading of aerosols in passenger cabins and compartments became a crucial evaluation parameter for air quality. Keeping in mind that one of the mayor transmission routes of the severe acute respiratory syndrome coronavirus type 2 (SARS-CoV-2) virus is airborne transmission via exhaled aerosols [1–4], social distancing, air purification devices and efficient ventilation strategies are promising control measures. Review articles e.g., by Kohanski et al. [5] and a correspondence letter by Morawska et al. [6] summarize the general status of current knowledge on the transmission of SARS-CoV-2. Herein, the suspension time, defined as the time a particle needs to settle from 1 m height, is given as high as 9.3 h for a 1 µm particle, whereas 10 µm particles settle within 5.6 min. In combination with the indoor air time for given air changes per hour (ACH), distinguishing whether the particles will settle on surfaces within the room or whether they are more likely to be removed by the ventilation system, is feasible. As a reference, the diameter of a SARS-CoV-2 virus itself is given as 0.1–0.2 µm. Further, the deposition fraction of inhaled particles for different parts of the breathing system is given as a function of the particle sizes. Summarizing, most of the particles with a diameter larger than 1 µm deposit in the nasal upper airway, whereas particles below 0.1 µm, are more likely to reach the alveolar system. Regarding the different exhalation modes, e.g., talking, breathing coughing or sneezing, it is concluded, that for all but sneezing, the majority of the particles is smaller than 10 µm and that the initial air exhalation flow is dissipated within less than 1 m from the mouth. From these finding, it is concluded, that the particles move mainly with the bulk air flow, which on the other hand is largely impacted by the thermal buoyancy of equipment and occupants as well as the forced air movement by the heating, ventilation, and air-conditioning (HVAC) system. Hence, the importance of the indoor local airflow pattern for COVID-19 transmissions is highlighted. For more detailed studies on the impact of different exhalation modes, e.g., breathing, talking and singing including a subdivision into categories of particle formation mechanisms (bronchial, larynx/trachea and oral) as well as an impact of the emitter’s age, the reader may be referred to [7, 8].

Within aircraft ventilation systems, high-efficiency particulate air (HEPA) filters and high cabin air flow rates [9] are already powerful measures for high standards in air quality level in terms of air pollution and also potential virus load. This is also confirmed by Rivera-Rios et al. [10] who report on lower aerosol concentrations in aircraft cabins compared to other indoor environments such as office rooms or restaurants. This is also confirmed by the fact, that the number of confirmed cases of transmission is aircraft cabins is rather low [11]. Nevertheless, it should be noted, that there are reported transmissions [12], underlining that there is an existing risk of transmission during flight.

The current state-of-the-art ventilation technique in passenger aircraft cabins is the so-called mixing ventilation. To ensure homogeneous conditions even for transient boundary conditions and different workload conditions, i.e., different number of occupied seats, the fresh air is supplied with rather high momentum below the luggage compartments and in the ceiling area [13]. The concept of high air supply momentum and the accompanying high amount of forced convection results in a strong mixing of the cabin air with the fresh air, and thus, guarantees the desired stable conditions. However, the high degree of mixing also enhances the potential transport of aerosols from one passenger to another. The quantification of this aerosol transport from one index passenger (the aerosol source) to other passengers, i.e., the direct transport, is addressed and analyzed in the present work.

Extensive and costly experimental investigations addressing the in-flight transmission risk of COVID-19-based data measured during flight-tests are given in Silcott et al. [14] and a report from the Netherlands Aerospace Centre (NLR) [15]. For selected aircraft types and predefined flight durations, they estimate mean infection risks in the range from 1/1800 to 1/120 and even up to 1/16 in case of super-spreader [15]. Here, fully occupied cabins (180–280 passengers, two class configuration) were considered and typical flight durations of 0.9–1.4 h and 8.7 h were assumed for the short-haul single-aisle and the long-haul twin-aisle flights, respectively. A mixture of 80% breathing and 20% speaking was considered and further, viral loads of 10^7.5^ virus copies per milliliter for a normal spreader and 10^10^ for a super-spreader were assumed following the values given in [17]. A dose–response relation, see also [17], was applied to calculate the infection risks.

In extensive numerical evaluations, Gupta et al. [18, 19] analyzed the spreading of aerosol particles in a 7-row aircraft cabin segment under state-of-the-art mixing ventilation. Their results proved that the ventilation system reduced the amount of actively flying aerosol particles 48, 32, 20 and 12% after 1, 2, 3 and 4 min, respectively. Further, they report that 3 min after the start of a continuous particle release, steady-state conditions were reached in their cabin segment. Based on these findings, they estimated the number of inhaled particles during a 4 h flight to be between 300 and 2000 under normal breathing conditions. Here, it should be noted, that not the number of the inhaled particles, but rather the amount of inhaled volume is relevant for the evaluation of infectious disease transmission.

With regard to alternative ventilation concepts for aircraft, You et al. [13] evaluate the spreading of contaminants by means of numerical tracer gas analysis. Their study, performed within two different 7-row cabin mock-ups, demonstrates an enhanced containment removal efficiency of cabin displacement ventilation over standard mixing ventilation, highlighting the importance and the chances of ventilation concepts to reduce airborne dispersion. Additionally, they propose a personalized ventilations system being a trade of between thermal comfort and contaminant removal efficiency.

A different approach to reduce the aerosol transmission from passenger to passenger is presented by Talaat et al. [16]. In their recent Reynolds-averaged Navier–Stokes (RANS) simulations study, they analyze the aerosol spreading by means of Lagrangian particle transport evaluation for three different configurations in a Boeing 737: a fully occupied cabin, empty middle seats of the seat benches and finally a configuration with ‘sneeze shields’ between the neighboring seats. Their results approve that the shields, which are approximately 34 × 74 cm^2^ (W × H) impermeable walls above the armrests, at a fully occupied cabin can reduce the transmission of aerosols below the level of a cabin with reduced occupancy. Further, their detailed results show the spreading of the aerosols is mainly located within a region of plus/minus two rows around the index passenger spanning both sides of the aisle.

In the present study, we address the aerosol dispersion in the passenger cabin of a regional aircraft. In that sense, it should be noted, that the current scientific knowledge on the SARS-CoV-2 transmission dynamics is not sufficient (yet) and strongly depending on the variants to evaluate a defined infection risk based on the aerosol dispersal measurements, see also Wang et al. [20] and the revision letter by Silcott et al. [14]. Nevertheless, the present results may a) be used by virologists as important input quantities for a downstream analysis of infection probabilities and b) highlight the potential of alternative ventilation concepts for a reduced aerosol dispersal in the cabin. However, the general statement: higher local aerosol contamination level (exhaled by an index passenger) results in higher potential infection risks should have validity.

Hence, we follow two main objectives in this paper: First, we want to experimentally determine the aerosol dispersion in a passenger aircraft cabin under state-of-the-art mixing ventilation and thus contribute to increase the knowledge on the current status. And second, we want to highlight the potential of alternative ventilation concepts for reduced aerosol dispersion within passenger cabins analyzing the results of RANS simulations.

## The Do728 test facility

**Fig. 1:**
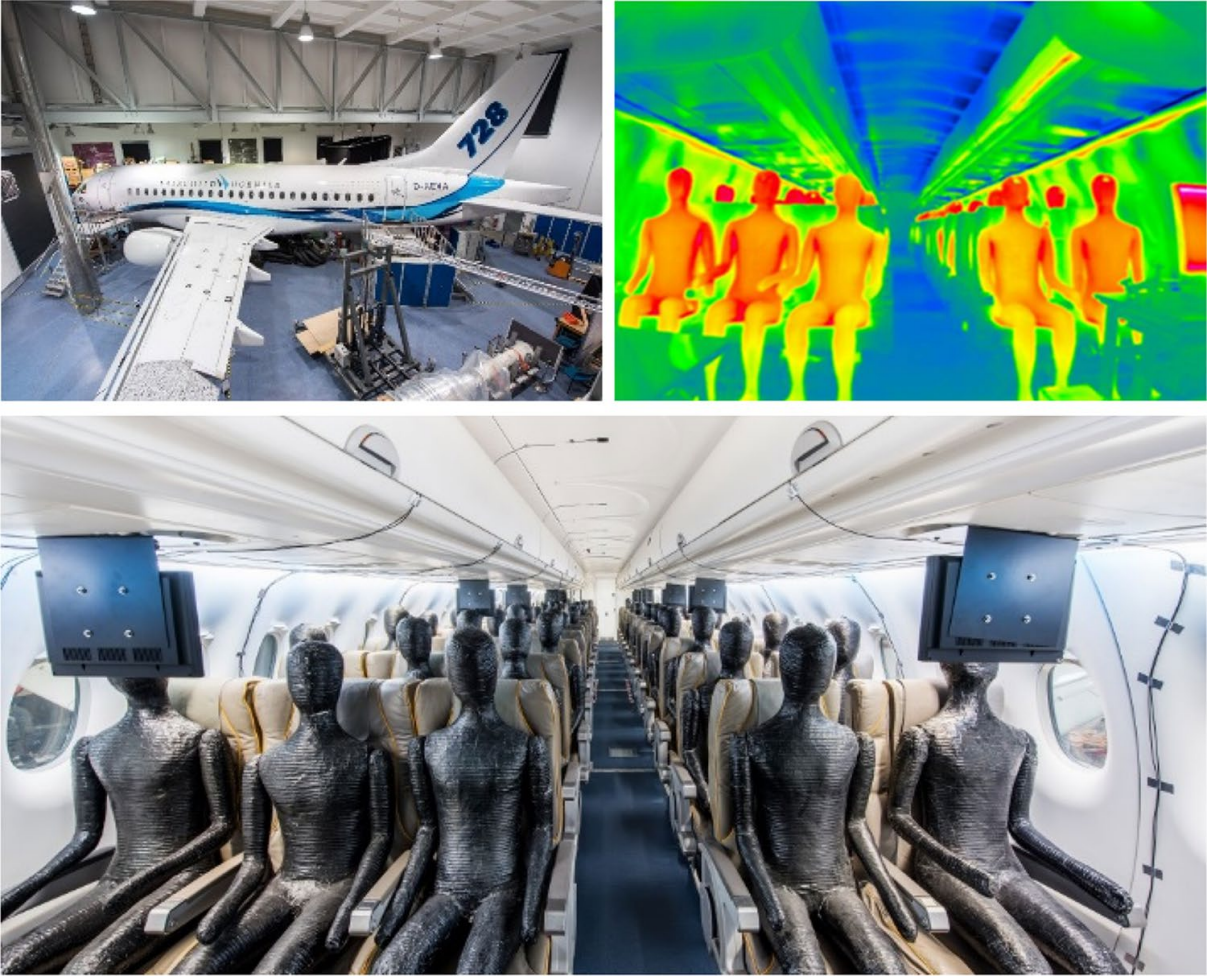
The Do728 research facility. Top left: exterior view, bottom: interior view with thermal manikins, top right: IR thermography with heated thermal manikins and cold air supply of mixing ventilation

The Dornier 728 (Do728) aircraft cabin test facility of the German Aerospace Center in Göttingen provides the opportunity for measurements in a real airplane without any certification efforts and with all other advantages of a stationary research facility. Furthermore, the Do728 comprises high flexibility for the integration of new technology bricks [21] and a plethora of latest measurement techniques are available for use. For a realistic reproduction of the cabin air flow, heated thermal manikins are used for the experimental simulation of the obstruction and the heat release of real passenger, see Fig. 1.

**Fig. 2:**
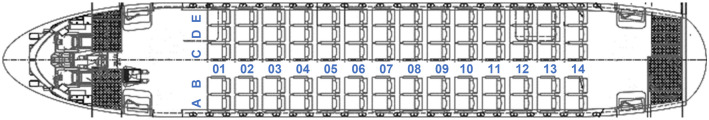
Seating layout of the Do728 including row and column labels in blue

The dimension of the cabin amounts to 3.25 m times 2.14 m times 14.5 m (W × H × L) and it is equipped with 14 seat rows of a three-two seating configuration, resulting in 70 seats in total, see Fig. 2. Details can be found in [21].

The air supply is realized using a stationary high-precision HVAC system in the basement of the experimental hall. To guarantee well-defined boundary conditions regarding the aerosol load of the supply air, the HVAC system is operated with 100% fresh air. Transferred to a real aircraft where fresh air and recirculated air are mixed, this assumption corresponds to a perfectly working HEPA filter. Accordingly, we investigate the dispersion of aerosols within the cabin and neglect the propagation of aerosols through the recirc system—which is expected to be very low due to the HEPA filter [9].

Final advantage of the Do728 research facility is, that a full computer-aided design (CAD) model optimized for computational fluid dynamics (CFD) simulations is available.

## Computational fluid dynamics for aerosol dispersion

The aerosol spread in the aircraft cabin is studied numerically using the open source OpenFOAM toolkit [22]. Although the study focuses mainly on the long-term dispersion of aerosol in the cabin, numerical modeling of the short-term process of aerosol cloud formation not only provides initial conditions, but is also of independent interest. The short- and long-term simulations differ from each other in the physical conditions of the simulated processes and therefore also request different numerical approaches. More precisely, when modeling the fast-passing initial cloud formation, it is necessary to consider the effect of particles on the flow, as well as humidity, temperature and velocity of exhaled air, the particle size distribution and evaporation of the aqueous components of the particles. On the other hand, the long-term spread of aerosol in the cabin is quite accurately modeled as a kinematic cloud propagation under the impact of gravity and drag forces.

We use standard steady-state and unsteady Reynolds-Averaged-Navier–Stokes (RANS and URANS, respectively) solvers applicable for weakly compressible to fully compressible, subsonic flows and the tools including the Lagrange-Eulerian approach implemented in OpenFoam for particle cloud simulations. In any case, the (U)RANS simulations here are performed with the k-omega SST turbulence model [23, 24] using the second-order accurate upwind numerical discretization schemes [25] for convective terms of any transport equation and second-order central schemes for the other terms.

Although, it is well known that the used k-omega SST turbulence model is not able to accurately model the effect of turbulent fluctuations in buoyancy driven flows, we use it due to its widely accepted good capabilities for pressure-driven flows. On the other hand, it is beyond the scope of the present study to adapt or develop a turbulence model capable of correctly predicting the physical processes evolving on turbulent mixed convection flows. As a consequence, the mean velocity fields provided by the below presented RANS simulations are reliable only for relative evaluation of three dimensional turbulent mean fields developing in the considered passenger cabins but not for the comparison of absolute velocity values.

For time integration in the URANS simulation, the first-order Euler implicit scheme is applied. The simulations are performed on a mesh with ~ 150 million grid points. The maximum cell size is 20 mm and the cells are refined approaching the walls such that the thickness of the wall cell layer is approx. 0.5 mm. The flow fields used as initial conditions URANS simulations are generated in a RANS simulation with the OpenFoam solver named ‘buoyantSimpleFoam’ [22] suitable for solving turbulent weakly compressible buoyant flows including radiation. Adiabatic thermal boundary conditions are used at all cabin walls, while the temperature of the incoming flow at the inlets is kept constant. Additionally, the thermal manikins emit an average heat flux of ~ 73 W.

### Initial particle cloud generation

The combined URANS and Lagrange–Euler simulations are performed with the unsteady OpenFoam solver named ‘reactingParcelFoam’, designed for subsonic, compressible turbulent flows with a multiphase particle cloud [22].

The particle injector is the ‘mouth’ of a thermal manikin characterized by an opening area of 1.45 cm^2^, similar to values in the literature [26]. The particles fly out in a stream of warm humid air (*T*_mouth_ = 35.5 °C, relative humidity > 80%) spreading in the surrounding dry air (relative humidity = 15%), which is typical in passenger aircraft cabins during cruise flight.

**Fig. 3:**
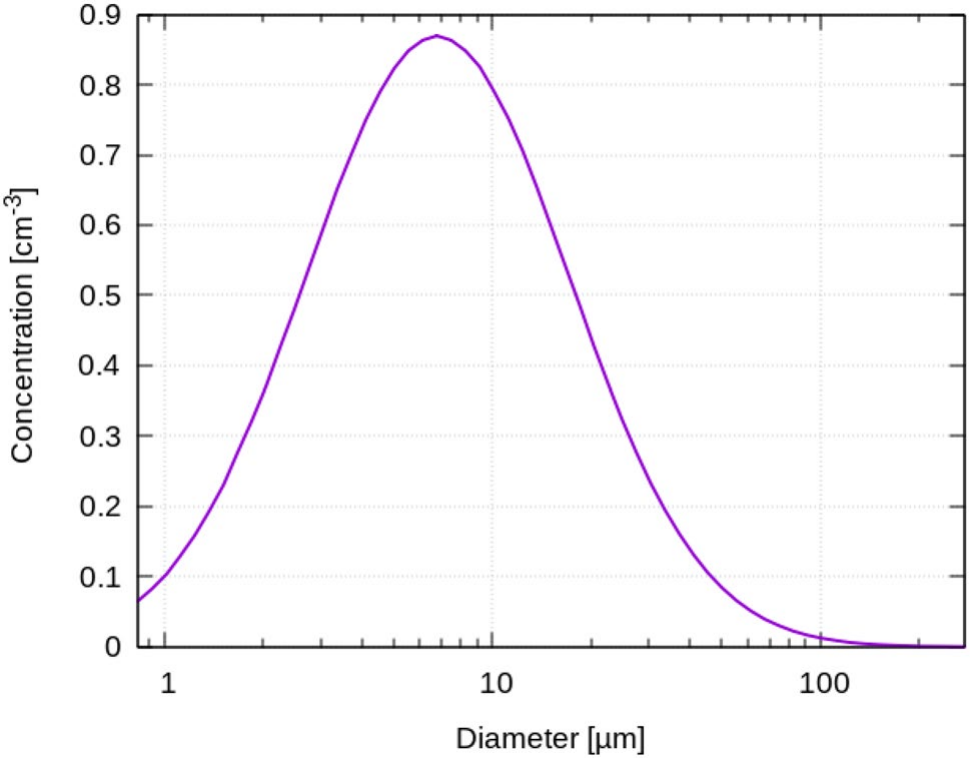
Distribution of initial particle diameter

The sizes of particles are distributed in a range of 0.8–275 µm according to log-normal distribution with median of 16 µm, see Fig. 3. Further, the particles contain liquid (H2O) and solid (C) components, which make up 94% and 6% of the initial volume, respectively, resulting in terms of initial mass to 88% and 12%. The assumed values for the mass and volume ratios are within the range given, e.g., in Bagheri et al. [7], wherein both, literature values and own measurements are summarized. They report for the shrinkage factors values ranging from 2.4 and 2.8 to 6.25 in the literature and approximately 4 in their own measurements. This means values for the solid compound from 7.2 to 0.4% regarding volume ratio, where our assumed value of 6% is at the upper end. Total volume of exhaled air when coughing is approx. 1 L, consequently peak velocities of 13.8 m/s are reached during the exhalation.

The high local particle concentrations (20,000 per liter) gives the volume fraction of ≈ 2 × 10^–6^, which means that following Elghobashi [27], a two-way coupling should be applied since the particles affect the surrounding air and vice versa.

**Fig. 4:**
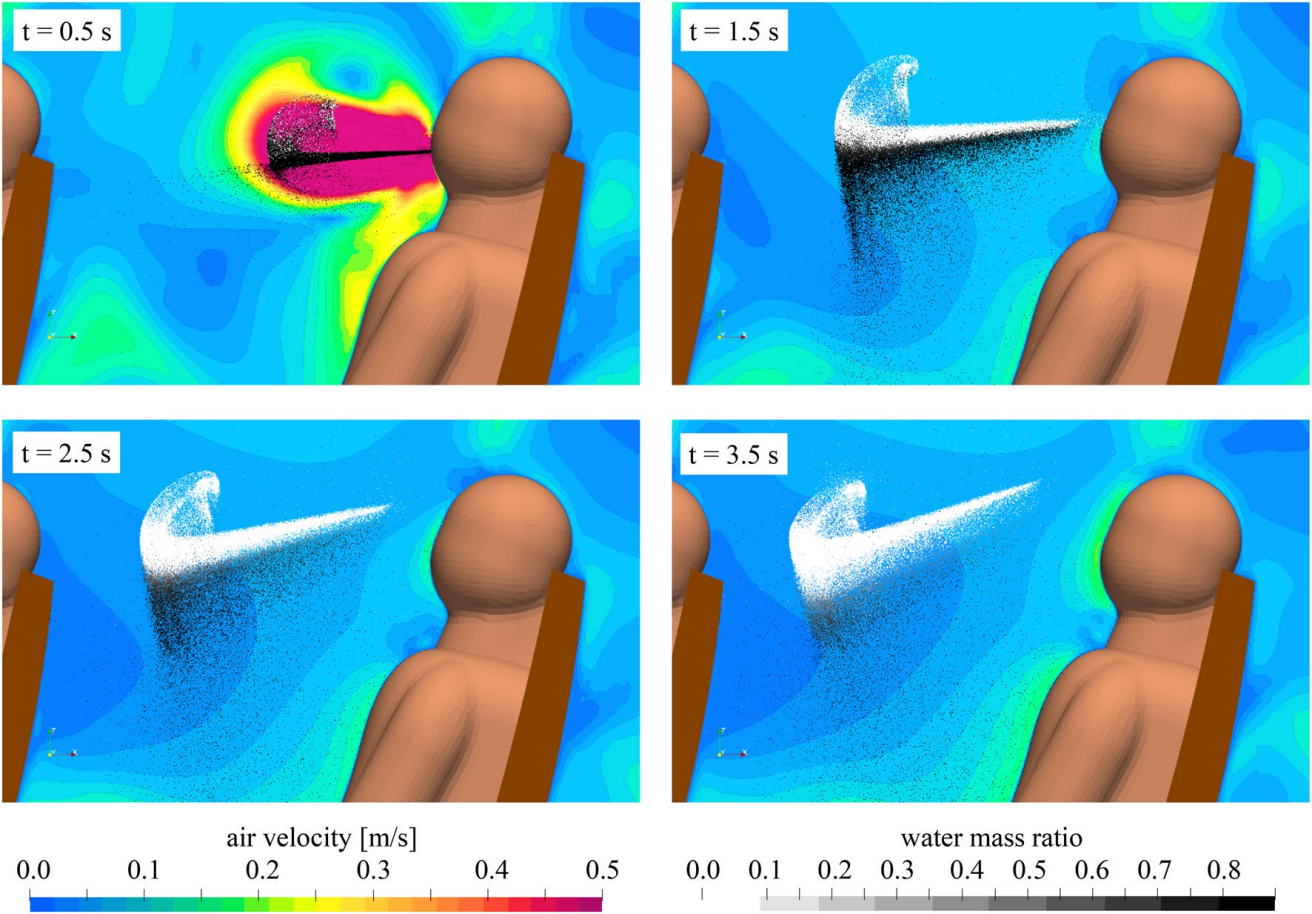
Aerosol cloud formation process for *t* = 0.5 s to *t* = 3.5 s after start the initial cough, predicted by the URANS simulations. Legends in the bottom apply for all images; color bar of air velocity is limited to dominant values, however, the peak velocity of the cough is 13.8 m/s

The URANS simulations reveal that within a short period of time (< 1 s after the end of the exhalation), small particles (initial diameter < 40 µm) lose their excess moisture, passing into an equilibrium state with the environment. These particles then form a long-lived aerosol cloud, while heavier droplets settle down on the surfaces. This separation is observed particularly in Fig. 4 (top right). Generally, looking at the instantaneous aerosol cloud formation process illustrated in Fig. 4, we find, that less than 4 s after the initial cough, two important mechanisms are already finished, (a) the liquid fraction of almost all expelled droplets is fully evaporated and only the solid core of the particles remain (see Fig. 4, bottom right) and b) the initial momentum of the cough event is fully degraded (see Fig. 4, bottom row). Consequently, the following two important assumptions allowing for cost-efficient RANS simulations are fulfilled (a) evaporation can be neglected and the final solid particles can be used and (b) the particle cloud for the dispersion analysis in the full cabin can be placed in the area in front of the index passenger, neglecting the exhalation momentum. The volume fraction of the particles in the resulting aerosol cloud, i.e., the total volume of all particles compared to the total volume of the aerosol cloud, is less than 10^–7^, so the particles no longer have a valuable effect on the surrounding flow [27]. This simplified essentially simulations of long-term aerosol spreading because it makes possible further to consider the aerosol cloud as a set of kinematic particles moving without interaction in the flow under the action of gravity and drag forces.

### Numerical dispersion modeling in the full cabin

The unsteady particle trajectories are determined based on the RANS flow fields using the Euler–Lagrange algorithm implemented in the OpenFoam solver named ‘icoUncoupledKinematicParcelFoam’ [22]. It exploits previously calculated steady velocity and turbulent kinetic energy fields. The latter is used to model stochastic the turbulent component of the velocity as proposed by Yuu et al. [28]. A log-normal distribution with a median of 5 µm and a width of 0.77 σ was taken as the initial particle size distribution for the visualizations and the determination of percentage of active/escaped/settled particles for different instants in time.

At this point, it should be noted, that we assume clean fresh air, i.e., 100% efficient filtering of the recirculated air, as also discussed in Sect. 2.

Highlighting the advantages of this statistical post-processing tool chain for the determination of the aerosol dispersion, we can summarize, that it is a very cost-efficient procedure allowing to analyze different source location and different diameter compulsions of the initial particle cloud. Thereby, the velocity field in the passenger cabin has to be determined only once and the Lagrangian particle propagation, i.e., the particle spreading analysis, is calculated during pure post-processing. As evaluation parameters, e.g., the concentrations in selected regions and the fraction of active/escaped/settled particles for different sized particles can be determined.

## Experimental aerosol dispersion methods

The experimental aerosol dispersion measurements contain two main tasks, the aerosol generation and the spatially resolved aerosol detection.

### Aerosol generation

**Fig. 5:**
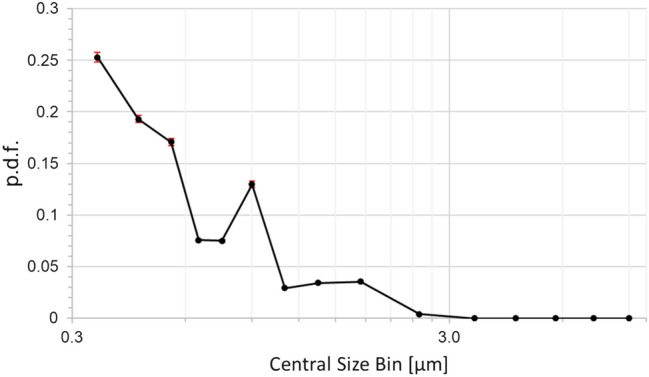
Distribution of experimentally released particle diameter

The aerosol generator consists of an airbrush pistol (AFC-101A, nozzle diameter 0.35 mm) pressurized at 2 bars and the volume flow is measured. It is used to disperse artificial saliva (mixed according to NRF 7.5^1^) [29]. Using this setup, a constant mist of dispersed saliva is provided. After the initial generation of the particles by the air brush nozzle, the particles are guided through a settling chamber. The setup of atomization chamber, settling chamber and pipe system is designed, that the particles have already an age of more than one minute before being released into the cabin, i.e., all evaporation processes are expected to be already finished, and pure dry particles are released. The particle size distribution of the experimentally generated particles is depicted in Fig. 5, showing, that particles with diameter smaller than 2.5 µm are produced corresponding, e.g., to normal breathing.

**Fig. 6:**
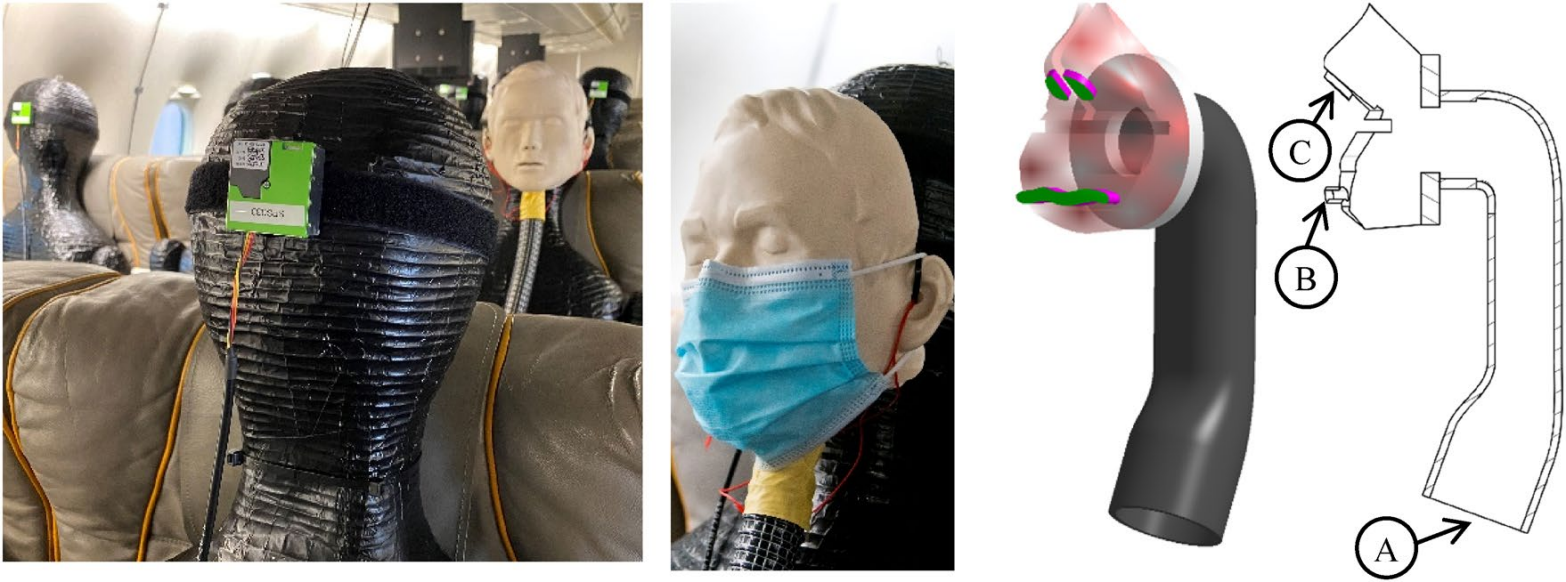
Left: Aerosol source (rear) and detection system (green sensors) both mounted on thermal manikins within Do728. Middle left: surgical mask attached to the aerosol exhalating manikin. Middle right: 3D Schematic drawing of the air ducts within the breathing face mask with exhaust areas marked in green. Right: 2D cross section of the breathing face mask with **A** connection to aerosol generator, **B** mouth and **C** nose openings

Moreover, the system can be connected to a facial mask (Fig. 6) to simulate realistic mouth-nose exhalation. The dimensions of the mouth and nose openings amount to $A_{{{\text{mouth}}}} \approx 3.5\;{\text{mm}} \times 35\;{\text{mm}} = 122\;{\text{mm}}^{2}$ and $A_{{{\text{nose}}}} \approx 2 \times 45\;{\text{mm}}^{2}$. A second high-pressure air supply valve with volume flow sensor is used for the control of the exhaust volume flow. An aerosol concentration sensor is integrated in the pipe system which connects the generator with the face mask. Consequently, the system allows a continuous monitoring of the aerosol generation rates, which amount to approx. 300.000 particles per second, to ensure a good signal to noise ratio during the measurements. The key facts of the aerosol source are summarized in a bullet point list:

- Aerosolization of artificial saliva (i.e., realistic liquids regarding thermodynamic properties)
- Particle sizes < 2.5 µm (e.g., breathing)
- Works with only pressurized air
- Volume flow rate and particle concentration probes integrated for continuous monitoring
- Defined aerosol production (quantity)
- Exhalation via realistic face geometry with mouth and nose openings
- Connected to thermal manikin

The last listed point, see also Fig. 6, ensures, that the aerosol source is a) geometrically in the right position and b) that the ‘index passenger’ is also a heat releasing thermal manikin, which is important for realistic cabin air flow fields and thus for a realistic aerosol particle dispersion.

### Aerosol detection

For the spatially resolved acquisition of particle number densities within the cabin 70 SPS30, low-cost particulate matter sensors are used [30]. Particles suspended in air scatter light originating from a laser diode inside the SPS30. The signal of a photo diode which measures the light intensity, is then internally converted to mass and number densities. A continuous air flow through the device transports fresh air into the measurement volume. An automatic self-cleaning mode ensures consistent measurement results during long-time measurements. The accuracy of this measurement device has been investigated by Tryner et al. [31]. They compare the results of low-cost particle matter sensors with the results of a larger direct mass transducer. With five different aerosol types they find the SPS30 being stable against changes in relative humidity and over long measurement periods, where the SPS30 outperforms the other sensors addressed in their study.

Additionally, a pre-calibration is performed using a high-precision optical particle sizer (TSI OPS 3330). Therefore, the aerosol particles are guided into a sealed box with the OPS probe and multiple SPS30 sensors inside. The pre-calibration confirmed that reliable results (i.e., error 10% of reading or 20/cm^3^ – whatever is larger) are obtained for two size bins: 0.3–1.0 µm and 1.0–2.5 µm. Details on the calibration procedure and the comparison of the OPS and the SPS30 results can be found in [32].

Afterward, the light-weight (26 g) and small (41 × 41 × 12 mm^3^) sensors are mounted at the face area of all seated thermal manikins, see Fig. 6, and thus the local aerosol concentration can be recorded on each seat.

The particulate matter sensors provide a sample rate of 0.9 Hz in our setup. Hence, the sensors are evaluating the average values within approx. 1 s of integration time, providing sufficient independent measurements a) for the discussion of the time development of the local aerosol concentrations and b) for the evaluation of the equilibrium state aerosol concentration, by calculating the time-average for each sensor over the last 300 s before switching off the aerosol source.

### Evaluation tool chain

For the evaluation of the experimental data, the following process tool chain is applied.

The first two steps (aerosol generation and detection) were already described in detail in the previous sections. After the local concentration data is recorded (step 2), we estimate the amount of potentially ‘inhaled’ aerosols (step 3). Therefore, we multiply the locally measured equilibrium-state particle concentrations [1/cm^3^] (time-averaging see Sect. 5.1.1) with the typical human tidal breathing volume (600 ml/breath) and with the typical breathing frequency of 10 breaths per minute [33]. Consequently, we end up with the amount of measured ‘inhaled’ particles per minute. Since our aerosol source produces much more aerosol particles compared to a breathing human, which is needed to increase the signal to noise ratio, we calculate in a step 4 the ‘inhalation fraction’. Therefore, we divide the rate of inhaled aerosols [particles/minute] by the rate of produced aerosols [particles/minute]. As a result, we obtain the number inhalation fraction (${f}_{N}={\dot{N}}_{\mathrm{seat}}/{\dot{N}}_{\mathrm{source}}$), which represents the amount of inhaled aerosol particles compared to the amount exhaled aerosols. Further, we calculate the volume inhalation fraction (${f}_{\mathrm{V}}={\dot{V}}_{\mathrm{seat}}/{\dot{V}}_{\mathrm{source}}$), which represents the volume of inhaled aerosol particles compared to the volume of exhaled aerosols. Due to the general applicability of this value ${f}_{V}$, we do not apply further steps in the evaluation tool chain in the presented results. In the Sects. 5.1.2–5.1.5, we use this fraction given in percent to analyze the aerosol dispersion in the passenger cabin.

For the sake of completeness, we would like to indicate an optional step 5 for ongoing evaluation. However, since at this point, the general applicability is reduced, we do not apply it within the present work. In this potential next step, the inhalation fraction should be multiplied with the number of aerosols or the virus quota emitted by an infected passenger per time unit. Further, a multiplication with the flight duration would result in the total number of inhaled aerosols or virus quota during the flight. In a final step, the knowledge of infection risk as a function of inhaled virus quota could result in spatially resolved infection risk maps. But, as a reminder, we evaluate our date ‘only’ toward the inhalation fraction, the optional steps could be performed by medicals or virologists based on our measured data.

## Results

The structure of the results section follows the two key questions raised at the end of the introduction: Sect. 5.1, focusses on the actual state of aerosol dispersion in a passenger aircraft cabin and Sect. 5.2 addresses the potential of an alternative ventilation concept. Thereby, the actual state of aerosol transmission is determined using experiments in the Do728 passenger cabin. In contrast, the alternative ventilation concept is evaluated based on RANS simulations with post-processing of the aerosol dispersion. The numerical approach also investigates the baseline case of mixing ventilation, for comparison with the alternative ventilation concept.

### Actual state (mixing ventilation)

**Fig. 7:**
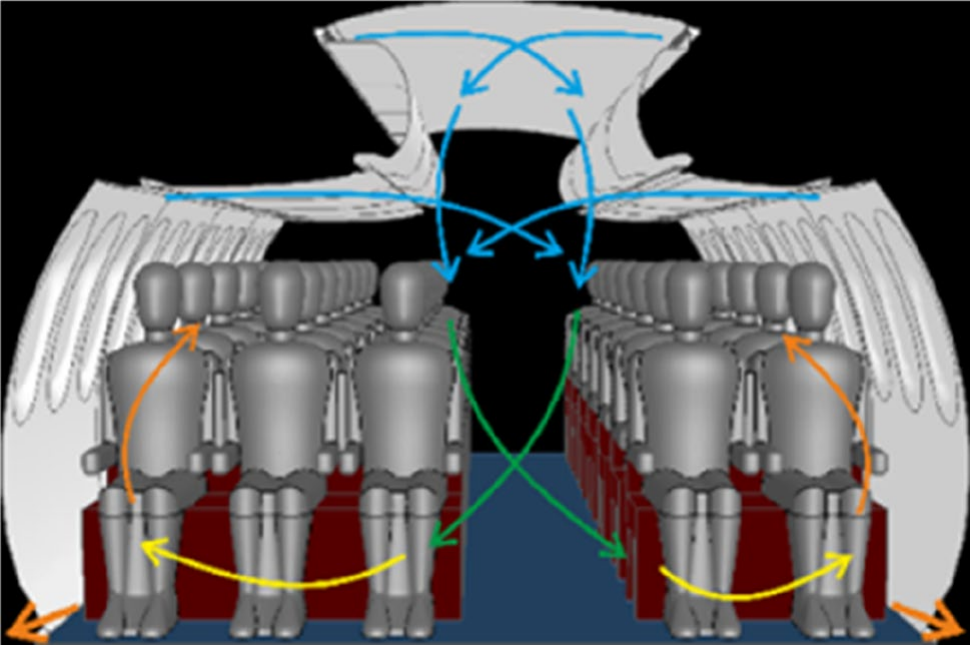
Sketch of state-of-the-art mixing ventilation (MV)

The experimental analysis presented in this paper concentrates on the actual state in aircraft cabins. Hence, we analyze the aerosol dispersion for state-of-the-art mixing ventilation where the fresh air is supplied through openings below the ceiling (ceiling air outlets — CAO) and below the luggage compartments (lateral air outlets — LAO). The Dado panels in the lateral foot room serve as exhaust openings where the air leaves the passenger cabin. A sketch of mixing ventilation is shown in Fig. 7.

#### Time development of the particle size-dependent aerosol concentration

The scope of this section is twofold, first, we discuss the time development of the particle size-dependent aerosol dispersion in the cabin based on the measured particle concentration–time data series for three selected seats in different distances from the aerosol source. Second, we briefly discuss the evaluation process for the discussion of the equilibrium state aerosol concentration described in the following sections.

**Fig. 8:**
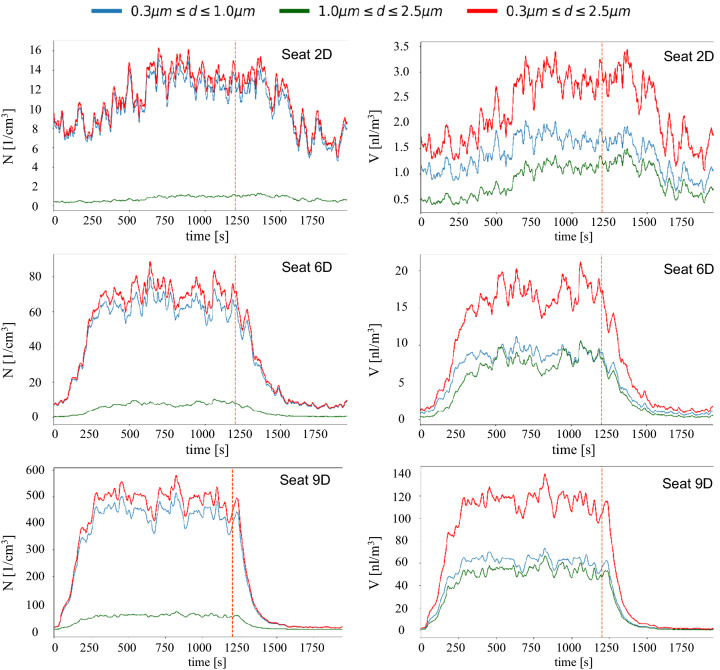
Experimentally recorded aerosol number–concentration (left) and volume aerosol concentration (right) on different seats during the course of one measurement run. Aerosol source started at t = 0 s; aerosol source stopped at dashed line. Aerosol source is located on seat 8E. Legend in the top applies for all images

In Fig. 8, we present the number–concentration (left) and volume–concentration in pico-liters per cubic meter (right) time series for three measurement points, seat 2D (top), seat 6D (middle) and seat 9D (bottom), for seat numbering, see Fig. 2. The aerosol source was located on seat 8E, i.e., the distance from the source increases from bottom to top. Please note, the different scales at the y-axis, ranging from 0 to 16 [1/cm^3^] (top), 0 to 90 [1/cm^3^] (middle) and 0 to 600 [1/cm^3^] (bottom) for the number concentrations and maximal volume concentrations ranging from 3.5 to 20 nl/m^3^ and 140 nl/m^3^. The different colored lines represent the different measurement bins, 0.3–1.0 µm (blue), 1.0–2.5 µm (green) and the sum of these two in red (0.3–2.5 µm). The aerosol source was started at *t* = 0 s and stopped at *t* = 1200 s (dashed vertical line).

Looking at the sum of all particle sizes (red lines), large differences regarding absolute values and the shape of the curves are found. Close to the source, the equilibrium value of approx. 500 particles per cm^3^ (120 nl/m^3^) was reached already after approx. 300 s and after the deactivation of the source, the values decrease to background level quickly in about 300 s, whereas the half-life *t*_1/2_ amounts to approx. 100 s. With increasing distance from the aerosol source, the absolute values of the equilibrium state decrease significantly and both, the incline as well as the decline curves are much less steep. Six rows in front of the source it takes approx. 700 s to reach the equilibrium state.

The comparison of the number–concentration (left) with the volume–concentration (right) highlights that the larger aerosol particles (green lines) have a negligible impact on the number–concentration whereas they contribute to up to 50% of the volume–concentration. Further away from the source (e.g., Seat 2D), the contribution of the larger aerosol particles decreases, but still reaches values of approximately 40% of the total aerosol particle volume.

A two-dimensional animation of the time-resolved aerosol dispersion (number–concentration) for the source position at seat 8E can be found in the video attached as supplemental material.

For the calculation of the equilibrium state aerosol concentration, which is afterward used in the evaluation tool chain discussed in Sect. 4.3, we calculated the time average for each sensor over the last 300 s before switching off the aerosol source. That is, the 300 s before the dashed orange line shown in Fig. 8. During this time period, only turbulent fluctuations of the concentrations are found around the local equilibrium values.

#### Influence of heat sources

In this section, we address the importance of realistic simulation of the passengers as one of many boundary parameters before diving into the analysis of the aerosol dispersion for different configurations. Thereby, the effect of the passengers’ thermal plumes on the exhaled particle trajectories was already documented by Yan et al. [34] based on computational fluid dynamics in a small cabin section with only three passengers.

**Fig. 9:**
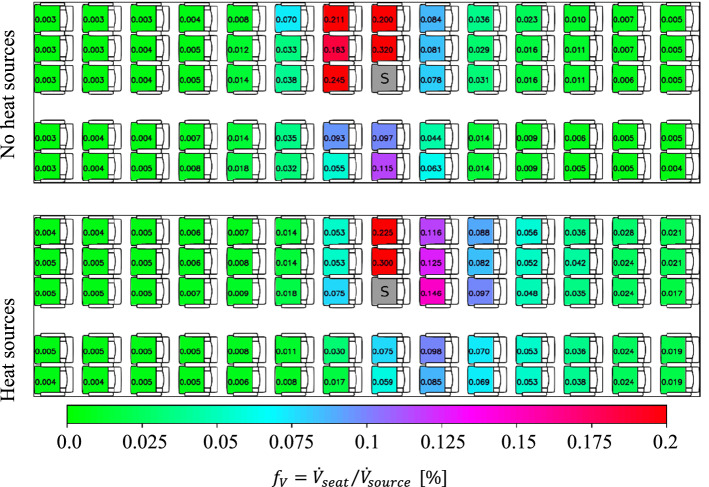
Spatial distribution of steady-state volume inhalation fraction for different passenger boundary conditions. Top: deactivated thermal manikins, i.e., no heat sources, bottom thermal manikins @75 W each, i.e., with heat sources. Aerosol source ‘S’ is placed at seat 8C. Legend in the bottom applies for all images

Figure 9 presents the spatial distribution of the volume inhalation fraction (see Sect. 4.3) evaluated in converged steady-state conditions, see also concentration–time series discussed in the previous section, without activated thermal manikins (top), i.e., the passenger dummies are not releasing any heat, and with activated thermal manikins (bottom). For the latter, the manikins are operated at a heat release rate of 75 W each, well corresponding to the sensible heat release rate of a typical seated human passenger. The breathing of the manikins was not simulated, except for the aerosol exhalating source manikin. We expect that this simplification has no significant impact on the overall flow field in the compartment since the tidal exhalation flow rate amounts only to about 1% of the HVAC supply air flow rate. This assumption is supported by the numerical results presented in Fig. 4, where the flow field is only affected within the fraction of a second during a cough event. The results of the tests with and without heat release of the manikins highlight two points, first, there is only a weak effect in the far-field (slightly increased values in the rear of the cabin). However, second, in the vicinity of the aerosol source, there are strong differences in terms of the aerosol load. Without heat sources, the dispersion is stronger in forward direction than in backward direction, likely caused by the momentum of the exhalation. In contrast, with heat sources, the aerosol dispersion is stronger toward the rear of the cabin. The heat release of the thermal manikins induces a thermal convection, which superimposes with the forced flow of the air supply system (and the exhalation). This superposition of forced and thermal convection induced large-scale flow structures in longitudinal direction in the passenger compartment leading to the changes in aerosol dispersion.

Consequently, for all results presented in the following, both experimentally and numerically, the thermal manikins are activated for a realistic flow pattern in the aircraft cabin.

#### Influence of the source location

**Fig. 10:**
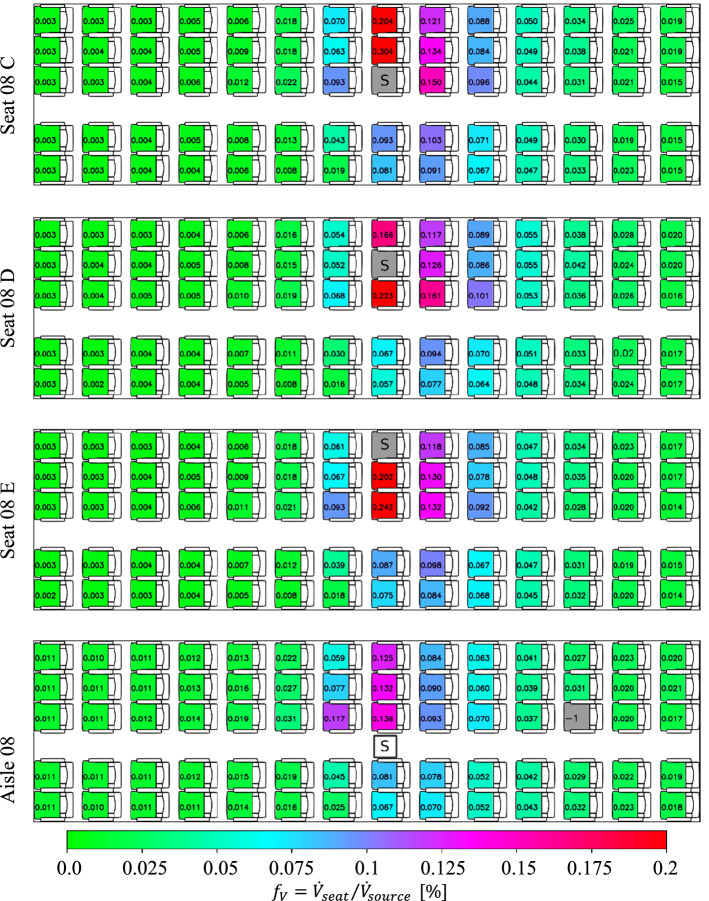
Spatial distribution of steady-state volume inhalation fraction for different source locations. From top to bottom: aerosol source (marked with a ‘S’) placed at seat 08 C, seat 08 D, seat 08 E and standing in the aisle in row 08. Legend in the bottom applies for all images. All manikins were heated at 75 W each

After this pre-discussion, we address the impact of the source location on the aerosol dispersion in this section. Therefore, the source was installed on different locations: three selected seats as well as standing in the aisle, i.e., at a height of 1.70 m, presented in Fig. 10. From top to bottom, the source is located on the aisle seat 08 C, the middle seat 08 D, the window seat 08 E and in the aisle in the row 08.

A comparison of the three different seat positions reveals that the highest peak load of ${f}_{\mathrm{V}}=0.30\%$ is found when the source is located on the aisle seat, decreasing to ${f}_{\mathrm{V}}=0.24\%$ (window seat) to ${f}_{\mathrm{V}}=0.22\%$ (middle seat). Otherwise, only minor differences between the local aerosol concentration values are found. For all cases, increased values above ${f}_{V}=0.06\%$ are only recorded less than two rows away from source. Also, the dispersion toward the other side of the aisle is only weakly depending on the source position, with the lowest values for the source on the middle seat.

In contrast, the dispersion from the standing source is much weaker compared to all seated source locations. Here both, peak values and overall load are strongly reduced. Most likely, this can be explained by the downward flow direction in the aisle enforced by the mixing ventilation air supply system. Thus, the exhaled aerosols are guided toward the floor and from there sideward to the exhaust openings.

#### Influence of a surgical mask

Following the analysis of the different source locations, we evaluate the effect of two rather simple counter measures, first the impact of putting a surgical mask on the aerosol source is discussed in this subsection.

**Fig. 11:**
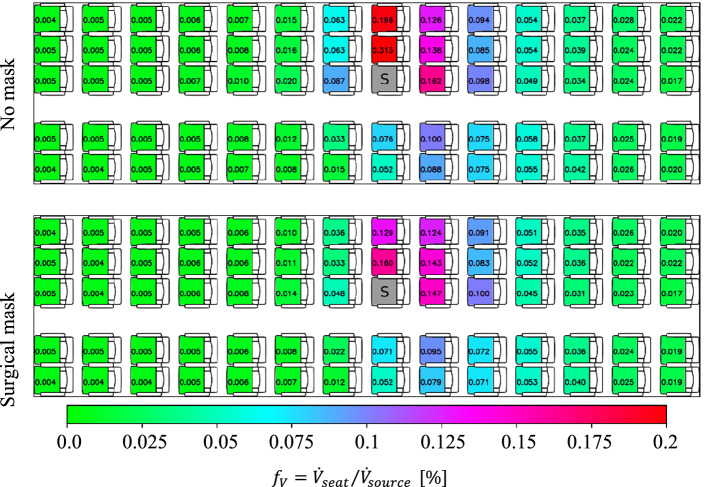
Spatial distribution of steady-state volume inhalation fraction for cases with and without a surgical mask. Top: no mask, bottom: surgical mask at source. Aerosol source placed at seat 8C (marked with a ‘S’). Legend in the bottom applies for all images. All manikins were heated at 75 W each

Here, it should be noted, that the surgical mask was not taped to the manikin, but normally fixed with the rubber bands behind the ears and adjusted to the nose of the manikin, see also Fig. 6. Following the explanation of Bagheri et al. [35] who measured at human subjects and Pan et al. [36] who measured at a manikin, the total outward leakage, i.e., the percentage of aerosol volume passing the mask (through the membrane and through leakages), increases from 0.25 to 0.6 for particle diameter decreasing from 2.0 to 0.5 µm. Figure 11 presents the effect of putting a surgical mask on the aerosol source. The baseline condition without a mask is presented in the top and the case with the mask in the bottom. Two main effects are found: First, the surgical mask reduces the peak load on the direct neighboring seat by approx. 50%. Second, the positive effect of the surgical mask is limited in forward and sideward direction (up to 50% reduction); however, rearwards no significant effect is found.

At this point we want to emphasize, that the mask was only applied to the aerosol source, i.e., the index passenger, in our experiments to analyze the effect on the aerosol dispersion in the cabin. Putting masks to the receiving passengers as well would lead to a further reduction of the inhaled aerosols by a factor which strongly depends on the type of mask and how well it fits the face, see e.g., [35] for an estimation of an upper bound of the infection risk for different masks and fitting in the face. Therein, Bagheri et al. evaluated the mean infection risk for different mask combinations on the infected and the susceptible showing, among others, that well-fitted FFP2 masks (at both persons) reduce the infection risk by more than 98% compared to surgical masks (at both persons). Even the combination with a well-fitted FFP2 mask at the susceptible and a surgical mask at the infected resulted in a reduction of the infection risk by about 85% compared to the case with both persons wearing surgical masks.

#### Influence of increased air flow rate

**Fig. 12:**
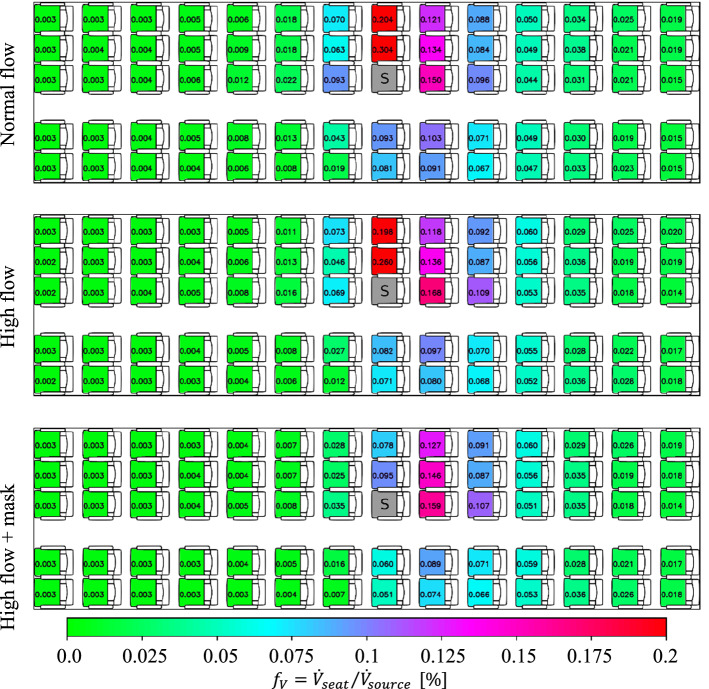
Spatial distribution of steady-state volume inhalation fraction for different air flow rates of the cabin ventilation system. From top to bottom: normal flow rate, high flow rate and combined high flow rate + surgical mask. Aerosol source placed at seat 8C (marked with a ‘S’). Legend in the bottom applies for all images. All manikins were heated at 75 W each

As second counter measure, we increased the air flow rate in the cabin by 10%. The results are depicted in Fig. 12 (middle). Additionally, the reference case is shown in the top of the image. The results reveal, that there is no change in the pattern of the aerosol dispersion, hence, the strengthening of the forced flow did not change the general flow pattern. As a reminder, the general flow pattern is a superposition of the forced and the thermal convection. Nevertheless, a precise look at the number values of the inhalation fraction reveals, that on most seats a slight reduction occurs for the increased high flow rate case. However, it should be noted, that on some seats, especially in the row direct behind the source, also increased values are found.

As last point, we investigated the combination of high flow and a surgical mask on the source, see Fig. 12 (bottom). Again, the effect behind the source is not significant. On the other side, a reduction of forward dispersion and an additional strong reduction within the row are found. Here most likely, both measures combine each other positively, i.e., the mask reduces the initial forward momentum of the exhalation and the higher flow rates suppress the dispersion within the row leading to up to 70% lower values on the corresponding seats.

### Alternative ventilation concepts

**Fig. 13:**
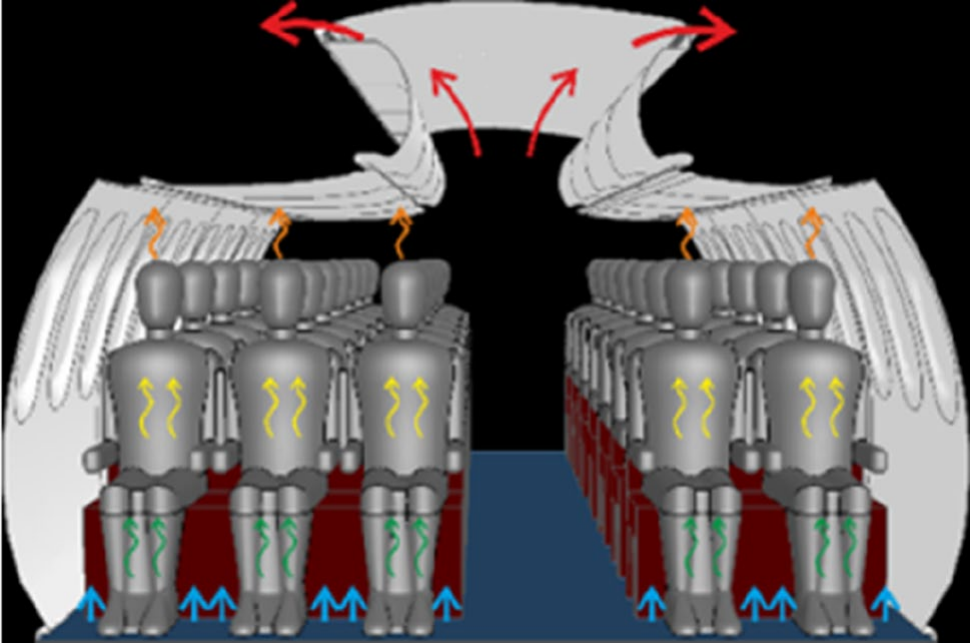
Sketch of cabin displacement ventilation (CDV)

To analyze the importance and the potential of the ventilation concept for an efficient removal of exhausted aerosols, RANS simulations are performed as described in Sect. 3. State-of-the-art mixing ventilation (MV), see Fig. 7, and cabin displacement ventilation (CDV), see Fig. 13, are studied as reference and alternative ventilation concept, respectively. The latter is based on low-momentum air supply on floor level and exhaust air openings in the ceiling area above the floor. Details on these concepts can be found e.g., in [13, 21].

**Fig. 14:**
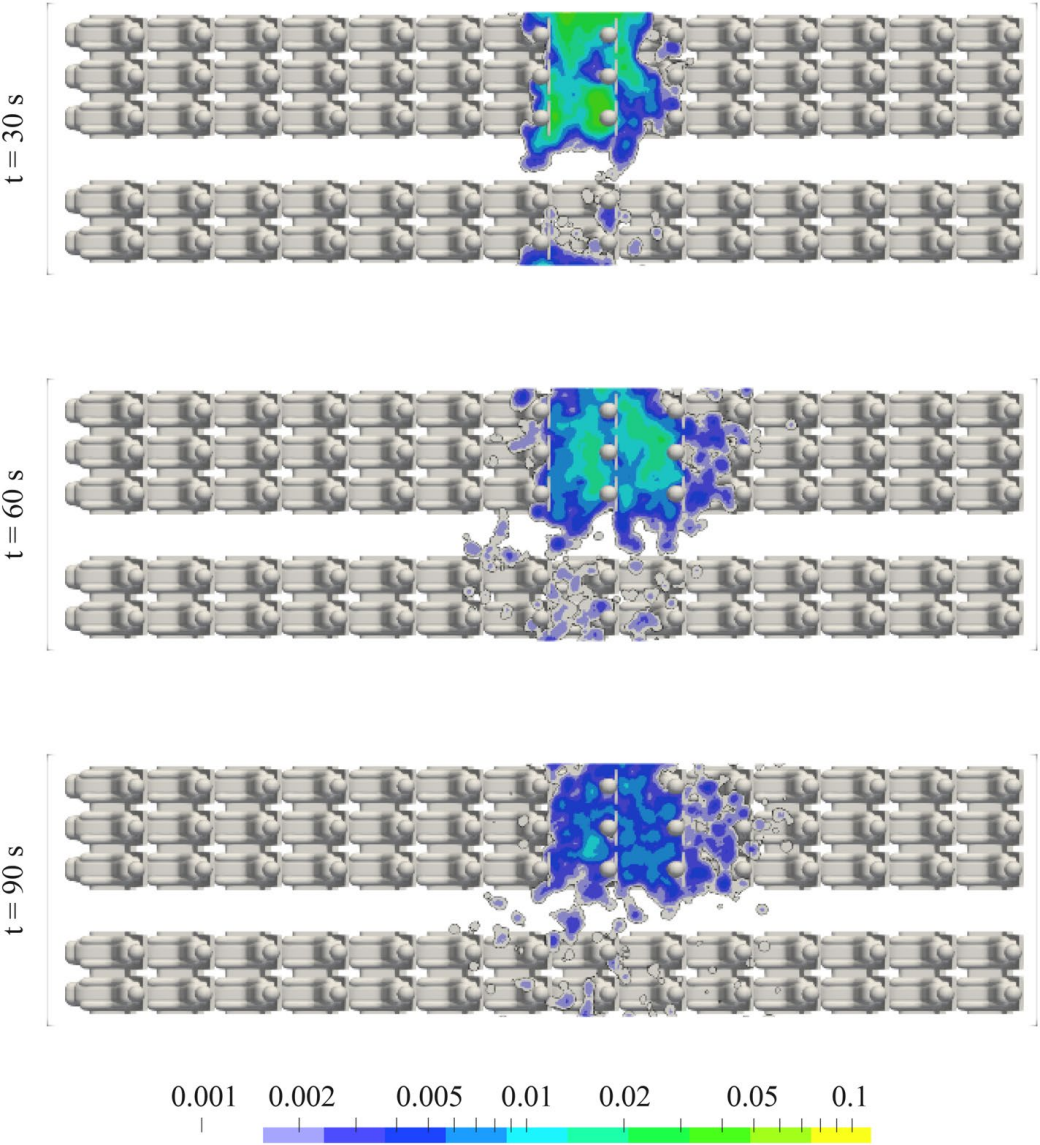
Aerosol concentration in the height of the breathing zone for MV @700 l/s at different instants in time predicted in RANS simulations. A concentration value of 1 corresponds to the initial aerosol concentration in front of the source at *t* = 0 s. Aerosol source on seat 8D. A log-normal distribution with a median of 5 µm and a width of 0.77 *σ* was taken as the initial particle size distribution. All manikins were heated at 75 W each

**Fig. 15:**
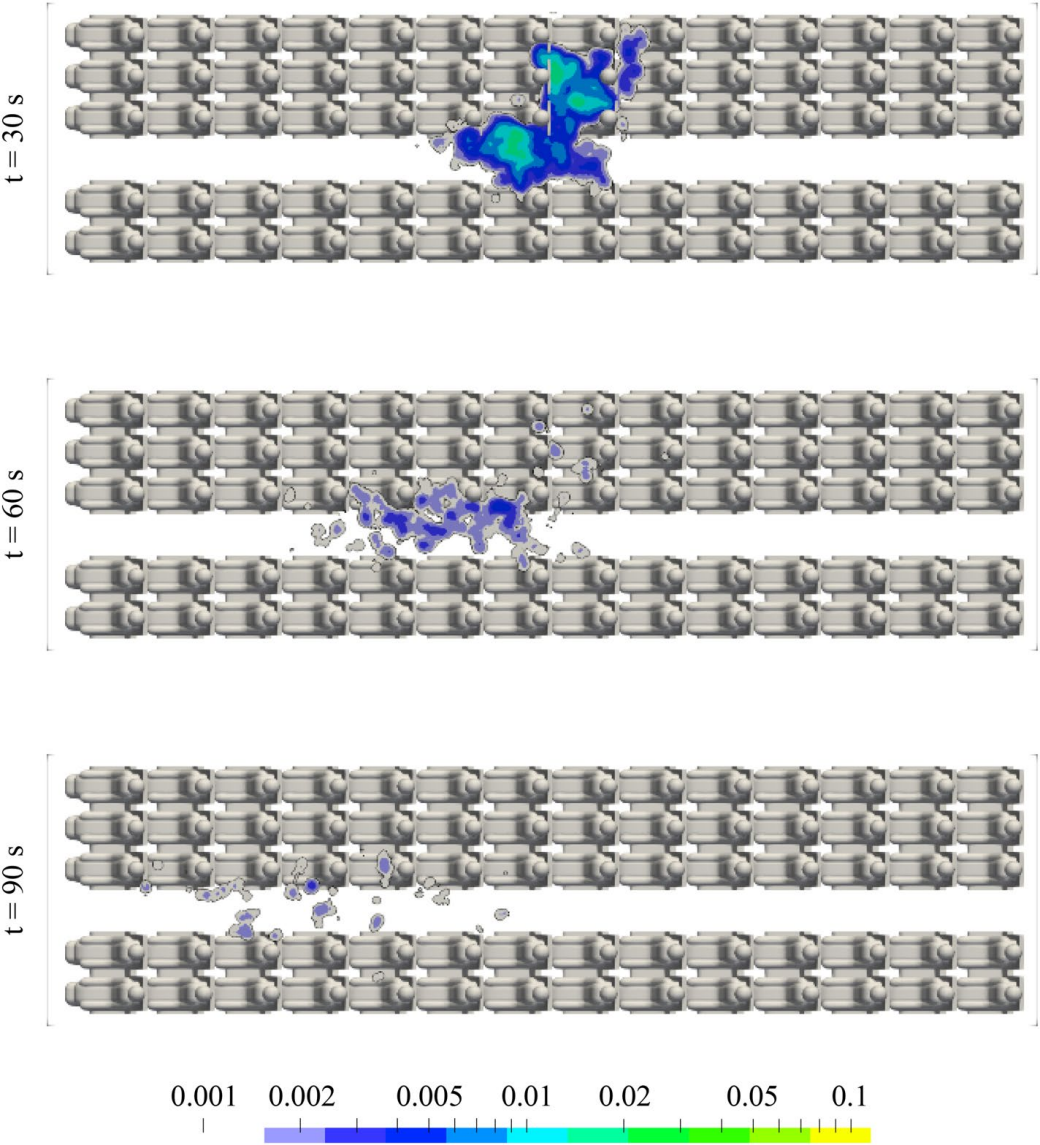
Aerosol concentration in the height of the breathing zone for CDV @900 l/s at different instants in time predicted in RANS simulations. A concentration value of 1 corresponds to the initial aerosol concentration in front of the source at *t* = 0 s. Aerosol source on seat 8D. A log-normal distribution with a median of 5 µm and a width of 0.77 σ was taken as the initial particle size distribution. All manikins were heated at 75 W each

Figures 14 and 15 show the spatial and temporal dispersion of aerosols starting from the central location of the three-seat row for MV and CDV, respectively, predicted in RANS simulations. Color-coded is the aerosol concentration in the breathing zone (0.97 m < *h* < 1.37 m), normalized to the initial concentration in front of the source. For mixed ventilation, aerosols are primarily distributed within the row of three and then in low concentrations also over the adjacent rows of seats and the other side of the aisle. This finding agrees to the experimental findings presented before. For CDV, in contrast to MV, the elevated aerosol concentrations firstly spread further to the front of the cabin, but then vanish quickly. A strongly reduced dwell time of the aerosols is found for CDV compared to MV.

**Fig. 16:**
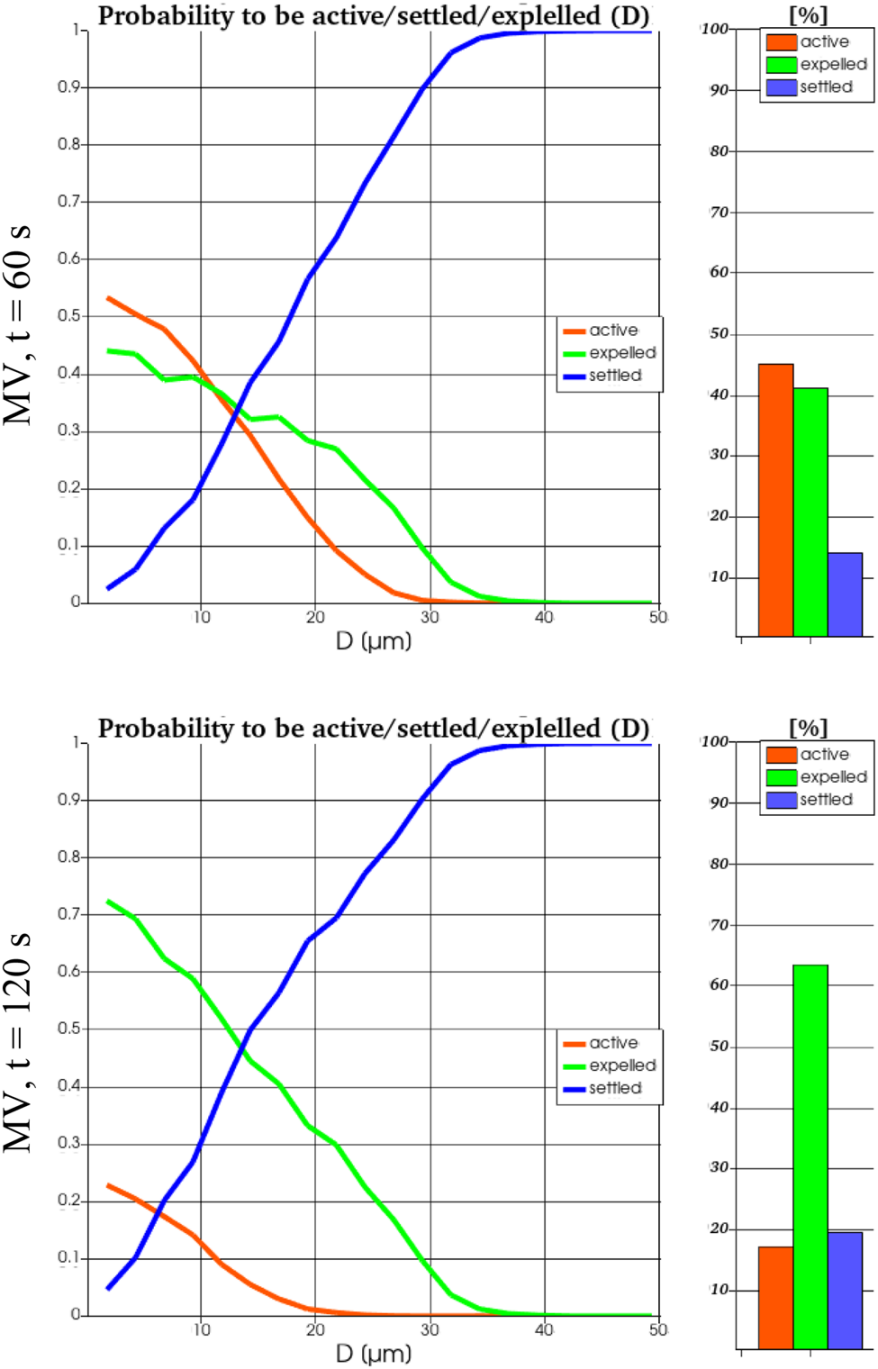
Ratio of still active (red), escaped (green) and settled (blue) particles for MV for two instants in time (top and bottom) obtained from RANS simulations. The histograms reflect the integrated values for all particle sizes of the log-normal distribution whereas the curves represent the size-dependent probability of a particle to be active, escaped or settled

**Fig. 17:**
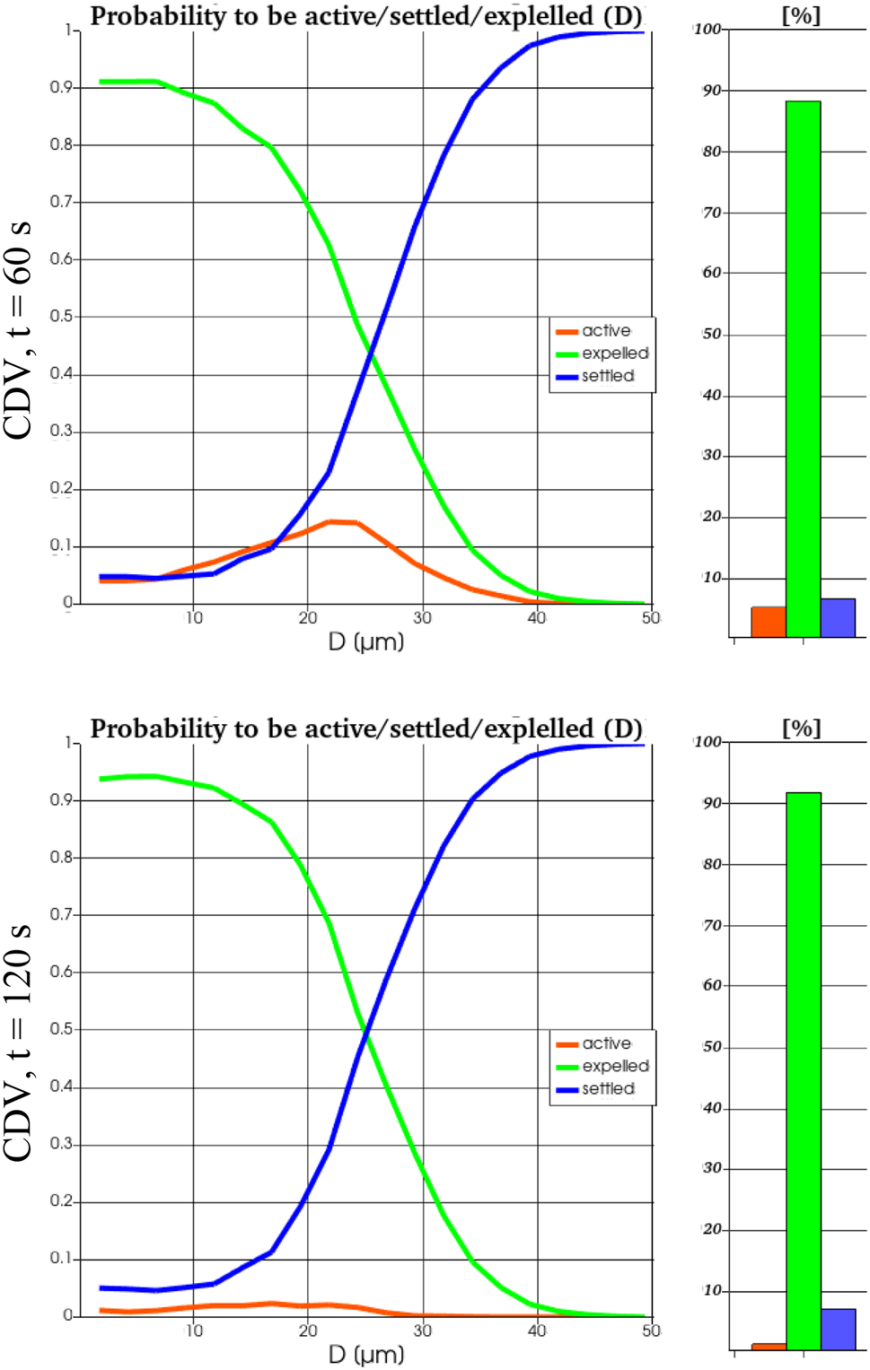
Ratio of still active (red), escaped (green) and settled (blue) particles for CDV for two instants in time (top and bottom) obtained from RANS simulations. The histograms reflect the integrated values for all particle sizes of the log-normal distribution whereas the curves represent the size-dependent probability of a particle to be active, escaped or settled

The histograms in Figs. 16 and 17, based the on numerical simulations, show the percentage of active (red), escaped (green) and settled (blue) aerosols for MV and CDV, respectively, and for two instants in time. Here, the above discussed log-normal distribution of the initial particle sizes was used for the evaluation of the numbers. 60 and 120 s after the particle injection, the remaining active particles in the cabin amount to 6% and ~2% for CDV, while at MV 45% and ~17% are still flying in the cabin, respectively. Additionally, the graphs in Figs. 16 and 17 present the size-dependent probability for a particle of diameter D to be active, escaped or settled. Main findings are, that the amount of settled particles (blue) is not changing between the presented instants in time anymore. For MV, the probability for all particles with D > 30 µm to be settled on a surface within the cabin or on another passenger is higher than 0.9 whereas for CDV it amounts only 0.75. Hence, larger particles settle faster for MV than for CDV. On the other hand, the probability to be escaped, i.e., removed by the ventilation system, is much higher for both instants in time and for all particle size for CDV compared to MV.

Most important, the probability of a particle to be active, i.e., still flying in the cabin, is time-dependent. At t = 60 s for MV it is much higher for particles with D < 20 µm, whereas for particles with D > 20 µm, it is higher for CDV. At t = 120 s for MV, still some small particles (D < 20 µm) can be expected to be active in the cabin. In contrast for CDV, the probability to be active is less than 3% for all particle sizes.

Concluding this short section on the investigation of alternative ventilation concepts, we can state, that a high potential to reduce the dispersion lengths and the dwell times exist. The local flow field in the cabin is the main parameter determining the dispersion of aerosols. Looking at CDV in specific, we confirmed the advantages over MV regarding the aerosol dispersion for all particles with D < 20 µm (final dry particle size). For larger particles, which might be generated, e.g., at uncovered coughing or sneezing, CDV revealed increased dwell-times compared to MV. Thereby, it must be noted, that the present particle spreading results are not directly transferable to sneezing events, as the initial momentum of these events cannot be neglected. The potential of alternative concepts, both, as redesign and as complete new development, should be considered when addressing the air quality for passenger aircraft cabins.

### Comparison of predicted and measurement particle concentrations

**Fig. 18:**
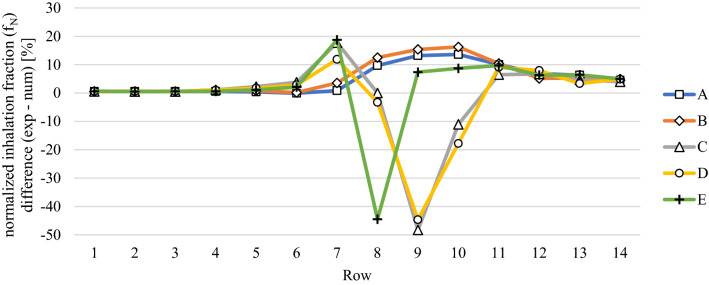
Comparison of measured (exp) and simulated (num) normalized inhalation fraction for the mixing ventilation case with high volume flow rate. The source was located on seat 8C. All manikins were heated at 75 W each

Finally, after discussing both, the experimental as well as the numerical analysis of the aerosol spreading in an aircraft cabin for state-of-the-art mixing ventilation and a novel ventilation concept, we will briefly compare the results from the numerical and the experimental approaches. As stated above, the predicted RANS velocity fields can only be used for evaluation of three dimensional turbulent mean fields. For the numerical estimation of an inhalation fraction, an inhalation volume (one liter) in front of the manikins’ mouths is defined. In the experiments, the sensors are directly placed at the thermal manikins’ faces and the test volume flow rate of the sensors (0.5 l/min) is lower than the normal human tidal breathing volume (6 l/min). Consequently, the sensors analyze the air directly at the mouths of the manikins and are not considering the inhalation of particles which might be a little further way. In conclusion, one cannot expect a perfect quantitative agreement between numerical and experimental analysis. For each technique solitary, relative changes, i.e., the comparison of cases can be evaluated with high reliability. Regarding the comparison of measured and simulated spreading behavior in the cabin, Fig. 18 depicts the inhalation fraction differences normalized to the maximum value of the numerical results, i.e., the percentage deviation between the two techniques. The values are presented for different columns (color-coded) and rows (x-axis). Spreading to the other side of the aisle (A and B) and toward the rear (rows 11 to 14) is stronger the in measured values compared to the simulated ones (positive values). However, close to the source (row 8) and on the same side of the source (D and E), the numerical values are larger than the experimental ones.

Finally, we want to emphasize that maximal deviations of 40–50% occur on three seats only. For more than 95% of the seats, the absolute deviations are smaller than 20% and still on 78% of the seats they are below 10%. Further, the mean absolute deviation amounts to as small values as 6.8%.

## Conclusion

We presented experimentally and numerically obtained results of aerosol dispersion in the Do728 passenger cabin. A realistic aerosol exhalation of artificial saliva through a facial geometry including mouth and nose openings was applied during the experiments and 70 aerosol-fine-particle sensors allowed for a space and time-resolved analysis of the dispersion. Firstly, we confirmed, that the heat release of the passengers is a crucial parameter when looking at local aerosol dispersion. Hence, all presented experimental and numerical analysis are performed with actively heated thermal manikins.

For state-of-the-art mixing ventilation, increased aerosol dispersion was found in an area covering one row in front and two rows behind the source. The main propagation takes place on the aisle side of the source. Locally, minor differences were found when the index passenger was moved from aisle to middle, to window seat. However, for a standing source in the aisle, strongly reduced aerosol dispersion was found. Further, the results confirmed the positive effect of a medical grade facial mask, which reduced the peak loads by about 50% (only the index passenger was wearing a surgical mask). Increasing the air flow rate in the cabin weakly reduced the peak loads.

In parallel, a cost-efficient computational fluid dynamics tool chain was developed to analyze aerosol dispersion using post-processing tools and pre-calculated flow fields. In the numerical study, cabin displacement ventilation as an alternative ventilation system was able to demonstrate advantages in terms of dispersion lengths and time residence of aerosols in the cabin for particles with D < 20 µm. However, increased dwell times of particles with D > 20 µm (dry final particle size) are found at CDV compared to MV. Regarding the total number of particles, 60 and 120 s after the particle injection, the remaining active particles in the cabin amount to 6% and ~2% for CDV, while at MV 45% and ~17% are still flying in the cabin, respectively.

Summarizing the key take-home messages from the experimental and numerical investigation:

- The value of potentially inhaled aerosols on the highest contaminated seat amounts to ${f}_{V}=0.31\%$ of the exhaled aerosols.
- Values of potentially inhaled aerosols ${f}_{V}>0.06\%$ of exhaled aerosols are only found within 4 rows around the index patient
- The ventilation system strongly affects dispersion

As also described in the evaluation procedure, we do neither estimate any infection risk for specific seats, nor a number of infections to occur during x-hour flight. The presented values of the rates of potentially inhaled aerosols as a fraction of the exhaled aerosols can be used as input for medicals and virologists to determine a (variant-dependent) infection risk. Nevertheless, the general statement: less aerosol contamination equals less infection risk will hold true for the interpretation of our results.

## Supplementary Information

Below is the link to the electronic supplementary material.

Supplementary file1 (MP4 3700 KB)

## Data Availability

Please contact the corresponding author for any data and material requests.

## Footnotes

1 According to NRF 7.5: 0.12 g KCl; 0.085 g NaCl; 0.25 g Na_2_HPO_4_* 12H_2_O; 0.015 g CaCl_2_* 2H_2_O; 0.005 g MgCl_2_* 6H_2_O; 0.1 g sorbic acid; 0.5 g carmellose sodium 400; 4.3 g sorbitol solution 70%; 1 g ad 100.0 mL H_2_O purify [29].

## Declarations

### Code availability

Please contact the corresponding author for any code requests.
